# Tackling the crisis in general practice

**DOI:** 10.1136/bmj.p966

**Published:** 2023-05-02

**Authors:** Carol Sinnott, Barbara Dorban-Hall, Mary Dixon-Woods

**Affiliations:** 1 THIS Institute, Cambridge, UK; 2 Cambridge University Hospitals, Cambridge, UK

## Abstract

Support to develop better systems will go a long way

The Health Foundation’s report on the Commonwealth Fund’s 2022 international health policy survey of primary care physicians in 10 countries makes for sobering reading.^1^ Illustrating some worrying trends, its standout findings include rising workloads, higher levels of burnout, and worsening job satisfaction. Strikingly, UK general practitioners score lowest of all countries in terms of satisfaction with the amount of time they spend with patients. Most (71%) find their work extremely or very stressful, with over (35%) of these GPs planning to stop seeing patients regularly in the near future. This level of extreme workplace stress and intention to withdraw poses severe threats to the sustainability of general practice. What solutions exist?

## Possible solutions

Some answers may lie in the Health Foundation’s report itself. Notably, GPs’ dissatisfaction does not seem, in the main, to relate to income. Instead, it is the increased volume and configuration of GPs’ workload, especially administrative work, that seems problematic. Administrative work is demanding, and the burden is compounded by the multiple operational failures routinely experienced by GPs in their daily work. Many of these relate to GPs’ role in coordinating care across multiple boundaries^2^ while often depending on incompatible systems and suboptimal communication. Individually, each operational failure—such as the rejected referral letter, the IT system that won’t load, and the delayed discharge information—may be small, but in aggregate they are time consuming, distracting, take time away from patients, and drain joy from work.^2^ ^3^ Evidence from other areas such as hospitals makes clear that apparently minor workplace dysfunctions make excessive contributions to stress and dissatisfaction.^4^

Reducing the administrative burden on GPs seems like an obvious target to help reduce stress and enhance wellbeing. Automation of routine tasks offers much promise,^5^ combined with opportunities to improve processes, workflows, communication, and supply chain management to make GPs’ administrative tasks less frustrating and time consuming. But the prospect of attempting this kind of system improvement at individual practice level is daunting: in the current context of high patient demand and staff shortage, the capacity available for improvement activity is very limited. 

Improvement efforts at individual practice level could also easily, and paradoxically, increase stress and other challenges. Each organisation painstakingly working out its own solutions without access to essential skills and support—such as technical skills in human factors and system design—is not an effective use of precious resources. Suboptimal design of improvement efforts, which is more likely when specialist expertise is lacking, could adversely affect patient experience and satisfaction with care. This was illustrated by the rapid transition to remote consulting during the pandemic—which, while necessary, caused difficulties for some groups.^6^ ^7^ As well as being wasteful, individual solutions result in lack of harmonisation across basic processes, introducing inefficiencies and threats to patient safety.^8^

## Learning system approach

A better solution is to tackle problems collaboratively and at scale through a learning system approach that includes patients and diverse staff groups.^9^ A primary care learning system could use routinely collected data to monitor care, understand problems, identify targets for improvement, co-design and develop prototype solutions, and implement and test changes with a view to improving both patients’ and GPs’ satisfaction.

Such an approach could take advantage of two key strengths of UK primary care. The first is the excellence of general practice data routinely collected by the NHS and high levels of GP data literacy. 1 Although security concerns, incompatible and outdated IT systems, lack of training in data coding and entry, and insufficient administrative and data analytics support will all need to be resolved,^10^ general practice data represent a key asset for improvement efforts. The second is the practice cluster and network infrastructure now in place in all four countries of the UK, which provides a way of coordinating and supporting improvement at scale.^11^ ^12^

These are challenging times for primary care. The causes are complex, and no single solution to the crisis exists. But supporting practices to achieve operational improvement is among the critical actions required to reverse the downward spiral in both patient and GP satisfaction, and reduce the extreme stress currently being experienced across primary care. 

## Notes

Provenance and peer review: Commissioned; not externally peer reviewed.
